# The Role of Fluorine-18-Fluorodeoxyglucose Positron Emission Tomography in Evaluating the Response to Treatment in Patients with Multiple Myeloma

**DOI:** 10.1155/2012/175803

**Published:** 2012-08-10

**Authors:** Carmelo Caldarella, Giorgio Treglia, Maria Antonietta Isgrò, Ivan Treglia, Alessandro Giordano

**Affiliations:** ^1^Department of Bioimaging and Radiological Sciences, Institute of Nuclear Medicine, Università Cattolica del Sacro Cuore, Largo Gemelli 8, 00168 Rome, Italy; ^2^Institute of Biochemistry and Clinical Biochemistry, Università Cattolica del Sacro Cuore, 00168 Rome, Italy; ^3^School of Biotechnology, Università Cattolica del Sacro Cuore, 00168 Rome, Italy

## Abstract

*Background and Aim*. Fluorine-18-fluorodeoxyglucose positron emission tomography (FDG-PET) is well recognized as a powerful diagnostic tool in the initial staging of patients with multiple myeloma (MM). The aim of this paper is to perform a systematic review about the usefulness of FDG-PET or PET/CT in evaluating the response to treatment in patients with MM. *Methods*. The scientific literature about the role of FDG-PET or PET/CT in evaluating the response to treatment in patients affected by MM was systematically reviewed. *Results*. Ten studies about the role of FDG-PET or PET/CT in evaluating treatment response in MM were retrieved and discussed. *Conclusions*. FDG-PET or PET/CT seems to be helpful in assessing the response to treatment in patients with MM and in the evaluation of possible sites of recurrent or progressive disease.

## 1. Introduction

Multiple myeloma (MM) is the second most common haematological malignancy. It accounts for <1% of all cancers and primarily affects older people, with a median age at diagnosis of about 65–70 years. The characteristic haematological alteration is monoclonal proliferation of plasma cells in bone marrow; in most cases, excessive production of monoclonal immunoglobulins can be observed and detected in serum and/or urine. Bone is involved in more than 80% of patients at the time of diagnosis, in most cases with evidence of osteolytic lesions; resulting pain, spinal cord compression, and hypercalcemia have a major impact on life quality [1–3].

The extension of bone marrow and extramedullary involvement should be carefully evaluated to establish prognosis and clinical management [4]. Currently, whole-body X-ray scan is the most commonly used diagnostic tool in the evaluation of bone involvement in patients with MM, due to the rare occurrence of extraosseous sites. Additional information could be provided by computed tomography (CT) and magnetic resonance imaging (MRI), while conventional bone scintigraphy is affected by low sensitivity because of inadequate osteoblastic activity in MM lesions [1–3].

Molecular imaging modalities such as Fluorine-18-fluorodeoxyglucose positron emission tomography (FDG-PET) or FDG-PET/CT have emerged in the recent years as useful methods in the initial staging and treatment planning of patients affected by MM.

FDG is a glucose analogue which is taken up by human cells by means transmembrane glucose transporters (GLUTs) and then phosphorylated to FDG-6-phosphate. Differently from normal glucose, it is not further metabolized to pyruvate and accumulates into the cytosol. The amount of intracellular FDG-6-phosphate is related to expression of GLUTs and, therefore, to glycolytic activity. Tumoral cells overexpress surface GLUTs and have a higher glycolitic activity than normal cells corresponding to an increased FDG uptake. However, intracellular uptake of FDG is not specific for neoplasms, because also activated macrophages in inflammation sites show an increased FDG uptake. In addition, the activation of haematopoiesis after antitumoral therapies could increase bone marrow FDG uptake diffusely. This explains why the assessment of eventual disease recurrence, persistence, or progression should not be performed shortly after the completion of chemotherapy cycles.

Evaluation of metabolic activity by using FDG-PET or PET/CT in suspected neoplastic lesions can be performed either qualitatively or semiquantitatively. Qualitative evaluation relies on the visual detection of focal sites of FDG uptake, whereas semiquantitative assessment is usually based on the calculation of standardized uptake value (SUV), which is a measure of glycolytic activity of the neoplastic lesions [3].

The role of FDG-PET and PET/CT in the initial staging of MM has been extensively investigated. These methods demonstrated a greater diagnostic accuracy compared to whole-body X-ray in diagnosing lytic bone lesions. Particularly, a recently published systematic review by van Lammeren-Venema et al. [3] has evaluated 18 studies on this topic. FDG-PET and PET/CT mostly showed more lytic lesions than conventional whole-body X-ray, excepted for skull lesions (perhaps because of the high metabolic activity of the brain); MRI seems to be less sensitive than FDG-PET or PET/CT, especially for the detection of extramedullary lesions, but more sensitive when the disease is diffusely spread through the spine [3, 5, 6].

In contrast with conventional imaging modalities, FDG-PET quantifies glycolytic activity of bone marrow lesions, thus allowing to distinguish nonactive and metabolically active lesions and to predict the risk of disease recurrence or progression after therapy. In fact, it has been demonstrated that several FDG-PET derived parameters, such as total disease burden or metabolic activity of lesions, play a significant role in establishing prognosis [7, 8]. Moreover, FDG-PET and PET/CT could help to identify eventual extraosseous sites of disease.

To date, few studies have investigated the role of FDG-PET or PET/CT in monitoring the response to treatment in patients with MM. Therefore, the purpose of this study is to perform a systematic review of the literature about the usefulness of this molecular imaging method in monitoring the response to treatment in MM.

## 2. Methods

A comprehensive computer literature search of the PubMed/MEDLINE, Scopus, and Embase databases was carried out to find relevant peer reviewed articles on the use of FDG-PET or PET/CT in monitoring the response to treatment in patients with MM.

A search algorithm based on a combination of the terms: (a) ‘‘PET” or ‘‘positron emission tomography” and (b) ‘‘myeloma” or “plasmacytoma” was used. No beginning date limit was used, and the search was updated until April 2012. To expand our search, references of the retrieved articles were also screened for additional studies. No language restriction was used. All studies or subsets in studies investigating the role of FDG-PET or PET/CT in evaluating treatment response in patients with MM or solitary plasmacytoma were eligible for inclusion.

The exclusion criteria were (a) articles not within the field of interest of this paper; (b) review articles, editorials or letters, comments, conference proceedings; (c) case reports. Two researchers (GT and CC) independently reviewed the titles and the abstracts of the retrieved articles, applying the inclusion and exclusion criteria mentioned above. The same two researchers then independently reviewed the full-text version of the articles to confirm their eligibility for inclusion.

For each included study, information was collected concerning basic study (author names, journal, year of publication, and country of origin), patient characteristics (number of patients and type of tumors evaluated), type of treatment, and device used and PET findings.

## 3. Literature Overview

The comprehensive computer literature search from the PubMed/MEDLINE, Scopus, and Embase databases revealed 226 articles. Reviewing titles and abstracts, 216 articles were excluded applying the criteria mentioned above.

Ten studies investigated the role of FDG-PET or PET/CT in monitoring the response to treatment in 690 patients with MM or solitary plasmacytoma: six of them have been conducted prospectively [9–14]; the remaining four studies have been conducted retrospectively [15–18]. These articles were selected and retrieved in full-text version. No additional study was found screening the references of these articles. The characteristics of the included studies are summarized in Table 1.

### 3.1. Prospective Studies

The usefulness of FDG-PET in monitoring response to treatment in MM was first investigated in 2002. Jadvar and Conti [9] conducted a prospective study of initial staging and posttreatment evaluation on 6 patients. Three patients were investigated with FDG-PET before and after treatment: in 2 patients, there was a decline in lesion metabolic activity, concordant with clinical improvement; the remaining patient, who encountered clinical deterioration after therapy, showed new hypermetabolic lesions and higher metabolic activity in the previously detected lesions. Other imaging studies showed no discernible changes.

In 2007, in a prospective study conducted by Zamagni et al. [10], posttreatment FDG-PET/CT scans of 23 patients, who have received autologous transplantation three months before, were compared with spine and pelvis MRI images. In 15 of them, posttreatment FDG-PET/CT showed reduction of metabolic activity, together with a marked improvement of immunoglobulin levels in 12 patients. Eight of these 15 patients had a normal MRI bone marrow pattern of spine and pelvis, whereas in the remaining 7 patients MRI was either unchanged or showed a reduced number of lesions. Eight patients showed no posttreatment improvement of FDG-PET/CT findings: six of them showed unmodified MRI bone marrow pattern in comparison with pretreatment findings.

The same authors have recently published a paper [13] in which 192 newly diagnosed MM patients were prospectively analyzed to demonstrate the prognostic relevance of FDG-PET/CT after thalidomide-dexamethasone induction therapy and double autotransplantation. Persistence of SUV greater than 4.2 in neoplastic lesions after induction therapy was an early predictor for shorter progression-free survival (44% versus 69% in patients with SUV values less than 4.2). Moreover, on multivariate analysis, incomplete suppression of FDG uptake after double autotransplantation was strongly associated with worst progression-free survival (32% versus 47%) and overall survival (66% versus 79%).

FDG-PET in monitoring response to treatment in patients with MM was prospectively evaluated on a wider population by Bartel et al. [11]. Baseline FDG-PET and MRI scans were performed in 239 patients and repeated after chemotherapy before autologous stem cell transplantation in accordance with “total therapy 3” program [19]. These authors found that the normalization of FDG uptake before autologous stem cell transplantation was an independent favourable prognostic variable. Conversely, persistence of PET positive lesions after therapy was inversely correlated to event-free survival (at 30 months from first autotransplantation, 89% of patients with posttreatment normalization of FDG-PET findings were event-free versus 63% of patients without suppression of FDG uptake).

Dimitrakopoulou-Strauss et al. [12] have investigated the prediction of progression-free survival in 19 patients with histologically confirmed MM, after anthracycline-based chemotherapy, by using dynamic FDG-PET. Median pretherapy SUV of the lesions was 2.24, while it was 1.74 after first cycle. Higher SUV values at baseline study were related to a shorter progression-free survival. The authors suggested that a full kinetic analysis of the FDG-PET studies performed at baseline and after chemotherapy could be helpful to predict progression-free survival and to identify patients who would benefit from anthracycline-based chemotherapy protocol.

Lastly, Derlin et al. [14] evaluated FDG-PET scans of 99 patients with MM, performed after autologous or allogenic stem cell transplantation, to assess the diagnostic performance of this diagnostic method for the detection and localization of residual or recurrent disease. Uniform response criteria [20, 21] were the reference gold standard: absence of monoclonal paraprotein in serum and urine and <5% of plasma cells in bone marrow were required to confirm complete clinical response. Overall sensitivity of FDG-PET for the detection of myelomatous lesions after stem cell transplantation was 54.6%, with a specificity of 82.1%, a positive predictive value of 82.3% and a negative predictive value of 54.2%. These results demonstrated the contribution of FDG-PET in localizing sites of active disease in patients with recurrent or progressive disease. Evidence of recurrence or progression on FDG-PET was associated with haematological recurrence or progression in all patients.

### 3.2. Retrospective Studies

The first retrospective analysis on this topic has been conducted by Mileshkin et al. [15]. In this study, 36 patients were investigated with FDG-PET for baseline assessment, restaging or suspected progression of disease. However, only six patients were further evaluated after radiation therapy. Negative findings on FDG-PET correlated with complete remission in 50% of patients; other three patients showed generalized disease progression on routine disease markers, but most previously irradiated lesions were not metabolically active. Furthermore, results of FDG-PET heavily impacted on management: particularly, in six cases the detection of progressive disease ensured the treatment plans were changed; in three cases, irradiation fields were widened to encompass active disease sites.

In 2005, Bredella et al. [16] have specifically investigated the value of FDG-PET in the assessment of bone marrow involvement in 13 patients with MM, demonstrating high sensitivity and specificity of this method in detecting myelomatous bone involvement. Additionally, 3 patients were studied with FDG-PET, conventional X-ray bone scan, CT and MRI before and after both chemotherapy and bone marrow transplantation, to assess the treatment response. Two patients showed a decline in metabolic activity after treatment and concordantly, clinical improvement; one patient developed recurrent disease, in a site distant to the original tumour. 

Kim et al. [17] have studied the impact of FDG-PET in 21 patients with apparently solitary plasmacytoma, for staging or restaging purposes. Fifteen patients had received definitive radiation therapy, but only 11 of them underwent posttreatment FDG-PET. Complete metabolic response was seen in seven patients, two patients developed progression to MM, and other two patients showed a partial metabolic response in the irradiated sites. However, late responses, up to 3 years after the treatment, were observed in these two latter cases. These results demonstrated that FDG-PET may be useful for staging and response assessment after radiation therapy in patients with solitary plasmacytoma, but a period of observation with serial imaging after therapy is required in some cases to assess metabolic response and to exclude new lesions.

Finally, Sager et al. [18] have correlated the FDG uptake of bone marrow lesions, in 42 patients with MM, to average bone marrow cellularity and plasma cell infiltration, calculated on a bone marrow biopsy specimen. Ten patients were referred for assessment of therapy response: FDG-PET/CT findings were negative in all except one patient, who showed metabolically active disease at one site. In nine patients, also posttreatment CT and MRI findings were negative and eight of them were histologically confirmed as being in remission; FDG-PET finding was false negative in one patient.

## 4. General Remarks and Conclusions

Our literature overview showed that FDG-PET or PET/CT could be helpful in monitoring response after treatment in patients with MM, mainly due to their ability to distinguish metabolically active lesions from inactive ones.

Notably, a baseline FDG-PET scan is mandatory as comparator to assess the treatment response at the posttreatment FDG-PET evaluation.

Furthermore, FDG-PET or PET/CT could detect the response to treatment earlier than other imaging methods such as whole-body X-ray and MRI, because functional changes assessed by FDG-PET usually precede morphological changes evaluated by conventional imaging methods.

Negative findings on posttreatment FDG-PET or PET/CT were mostly correlated with complete clinical and histological remission or, at least, low risk of recurrences or disease progression; persistence of metabolically active lesions was related to shorter overall and event-free survival. Therefore, posttreatment FDG-PET findings could be of higher prognostic significance than standard response monitoring methods.

Unfortunately, to date, the response to treatment using FDG-PET or PET/CT and the prognostic value of these techniques have been mostly evaluated on a small number of patients with MM, and further large and multicentric prospective studies are needed to substantiate the role of FDG-PET or PET/CT in this setting.

Moreover, qualitative or semiquantitative criteria used to define the positivity of a FDG-PET scan for recurrence or disease progression were not clearly specified in most papers.

Interestingly, we included a paper about usefulness of FDG-PET in patients with solitary plasmacytoma [17], showing that this technique might also be useful to confirm or exclude progression to MM and to assess complete metabolic response after radiation therapy. However, late responses should be taken into account.

In conclusion, it should be expected that, in the near future, FDG-PET or PET/CT will be used even more in the assessment of metabolic response after treatment in patients with MM, as a guidance for clinical decision and to eventually decide for alternative therapies in nonresponding patients.

## Figures and Tables

**Table 1:** Characteristics of the studies using FDG-PET or PET/CT to monitor treatment response in multiple myeloma.

Authors	Year	Country	Study design	Diagnosis	Treatment (no. of patients)	No. of patients	Device	Posttherapy FDG-PET findings		Complete remission
								Persistence of FDG uptake	Normalization of FDG uptake	
Jadvar and Conti [9]	2002	USA	Prospective	MM	CRT (2) CT + BMT (1)	3	PET	2	1	1
Mileshkin et al. [15]	2004	Australia	Retrospective	MM	RT	6	PET	3	3	ns
Bredella et al. [16]	2005	USA	Retrospective	MM	CRT + BMT	3	PET	1	2	2
Zamagni et al. [10]	2007	Italy	Prospective	MM	CT + BMT	23	PET/CT	8	15	12
Bartel et al. [11]	2009	USA	Prospective	MM	CT	239	PET	ns	ns	ns
Kim et al. [17]	2009	USA	Retrospective	PLC	RT	11	PET	4 (^∗^)	7	9 (^∗∗^)
Dimitrakopoulou-Strauss et al. [12]	2009	Germany	Prospective	MM	CT	19	PET	ns	ns	ns
Sager et al. [18]	2011	Turkey	Retrospective	MM	CT	10	PET/CT	1	9	8
Zamagni et al. [13]	2011	Italy	Prospective	MM	CT (85)	85	PET/CT	54 (improvement in 12)	31	ns
					CT + BMT (192)	192		67 (improvement in 32)	125	ns
Derlin et al. [14]	2012	Germany	Prospective	MM	BMT	99	PET/CT	ns	ns	40

Legend—ns: not specified; CT: chemotherapy; RT: radiation therapy; CRT: chemoradiation therapy; BMT: bone marrow transplantation; MM: multiple myeloma; PLC: solitary plasmacytoma; (^∗^): evolution to MM in 2 patients; (^∗∗^): late responses in 2 patients.
